# Secondary Metabolite Transcriptomic Pipeline (SeMa-Trap), an expression-based exploration tool for increased secondary metabolite production in bacteria

**DOI:** 10.1093/nar/gkac371

**Published:** 2022-05-17

**Authors:** Mehmet Direnç Mungan, Theresa Anisja Harbig, Naybel Hernandez Perez, Simone Edenhart, Evi Stegmann, Kay Nieselt, Nadine Ziemert

**Affiliations:** Interfaculty Institute of Microbiology and Infection Medicine Tübingen (IMIT), University of Tübingen, Auf der Morgenstelle 28, 72076 Tübingen, Germany; Interfaculty Institute for Bioinformatics and Medical Informatics (IBMI), University of Tübingen, 72076 Tübingen, Germany; German Center for Infection Research (DZIF), Partnersite Tübingen, 72076 Tübingen, Germany; Interfaculty Institute for Bioinformatics and Medical Informatics (IBMI), University of Tübingen, 72076 Tübingen, Germany; Interfaculty Institute of Microbiology and Infection Medicine Tübingen (IMIT), University of Tübingen, Auf der Morgenstelle 28, 72076 Tübingen, Germany; Interfaculty Institute of Microbiology and Infection Medicine Tübingen (IMIT), University of Tübingen, Auf der Morgenstelle 28, 72076 Tübingen, Germany; Interfaculty Institute of Microbiology and Infection Medicine Tübingen (IMIT), University of Tübingen, Auf der Morgenstelle 28, 72076 Tübingen, Germany; German Center for Infection Research (DZIF), Partnersite Tübingen, 72076 Tübingen, Germany; Interfaculty Institute for Bioinformatics and Medical Informatics (IBMI), University of Tübingen, 72076 Tübingen, Germany; Interfaculty Institute of Microbiology and Infection Medicine Tübingen (IMIT), University of Tübingen, Auf der Morgenstelle 28, 72076 Tübingen, Germany; Interfaculty Institute for Bioinformatics and Medical Informatics (IBMI), University of Tübingen, 72076 Tübingen, Germany; German Center for Infection Research (DZIF), Partnersite Tübingen, 72076 Tübingen, Germany

## Abstract

For decades, natural products have been used as a primary resource in drug discovery pipelines to find new antibiotics, which are mainly produced as secondary metabolites by bacteria. The biosynthesis of these compounds is encoded in co-localized genes termed biosynthetic gene clusters (BGCs). However, BGCs are often not expressed under laboratory conditions. Several genetic manipulation strategies have been developed in order to activate or overexpress silent BGCs. Significant increases in production levels of secondary metabolites were indeed achieved by modifying the expression of genes encoding regulators and transporters, as well as genes involved in resistance or precursor biosynthesis. However, the abundance of genes encoding such functions within bacterial genomes requires prioritization of the most promising ones for genetic manipulation strategies. Here, we introduce the ‘Secondary Metabolite Transcriptomic Pipeline’ (SeMa-Trap), a user-friendly web-server, available at https://sema-trap.ziemertlab.com. SeMa-Trap facilitates RNA-Seq based transcriptome analyses, finds co-expression patterns between certain genes and BGCs of interest, and helps optimize the design of comparative transcriptomic analyses. Finally, SeMa-Trap provides interactive result pages for each BGC, allowing the easy exploration and comparison of expression patterns. In summary, SeMa-Trap allows a straightforward prioritization of genes that could be targeted via genetic engineering approaches to (over)express BGCs of interest.

## Graphical Abstract

**Graphical Abstract:**
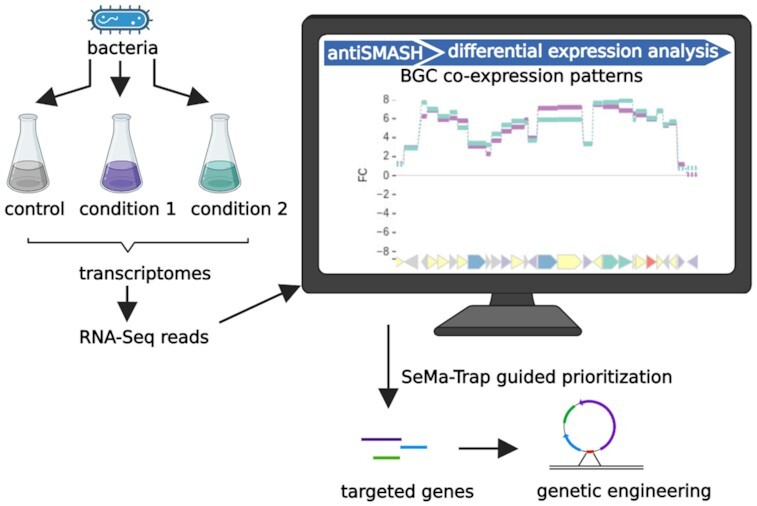
Secondary Metabolite Transcriptomic Pipeline (SeMa-Trap), an expression-based exploration tool for increased secondary metabolite production in bacteria.

## INTRODUCTION

By providing a wide range of biological functions, natural products have been foundational to the survival and evolutionary fitness of various organisms in the tree of life (1). Also known as secondary metabolites (SMs), these compounds are abundantly produced by plants and microorganisms (2). For decades, these molecules have been fueling various industries such as pharmaceutics as antimicrobial agents (3,4). However, the decrease in the discovery rates of novel antibiotics and the parallel increase in resistance towards the existing antibiotics make the identification of new bioactive compounds a task of paramount importance (5). By encoding the enzymes necessary for compound production, biosynthetic gene clusters (BGCs) represent the organized groups of genes involved in the production of SMs (6). During the last decade an enormous number of genomic sequences have been made available, revolutionizing genome mining efforts in natural product research (7). Based on algorithmic concepts like hidden Markov models (HMMs), highly improved computational tools for BGC prediction such as antiSMASH (8) enable rapid mining of sequenced genomes. By using such tools, thousands of BGCs have been made available to researchers stored in public databases such as MIBiG (9), antiSMASH-DB (10) or The Natural Products Atlas (11). However, from the entire bacterial kingdom, it was recently shown that only 3% of its genomic potential for SMs has been experimentally verified (12). One of the main reasons for this phenomenon is that the expression of the BGCs is often tightly regulated and not observed under laboratory conditions. This non-expressed nature of the BGCs creates a major bottleneck in the identification of bioactive compounds with novel modes of action (13).

To activate silent BGCs and increase the production titers of SMs, several strategies have been devised such as altering the culturing conditions or heterologous expression of the BGCs (14,15). Additionally, genetically modifying global and local regulatory genes can enhance transcription levels of biosynthetic genes (16). Activation or disruption of positive and negative regulators, respectively, has led to the expression of many silent BGCs (17,18). Furthermore, it has been shown that increasing the expression of genes encoding transporters (19), conferring resistance (20), or involved in precursor supply (21) also increases SM production. However, major antibiotic producers like the organisms belonging to the genus *Streptomyces* (22) encode around 7000 genes on average (23). This raises the question: Which ones to genetically modify? Comparative transcriptomic analyses based on RNA-sequencing (RNA-seq) can help decipher the complex pathways that regulate the BGCs of interest and thereby, select the genes to prioritize (hereinafter referred to as target genes) (24,25). This strategy is mostly conducted by comparing the expression levels of BGCs from organisms with genetic variance or from the same strain cultured under different physiological conditions (26,27). The overwhelming number of possible experimental designs make the prioritization of promising culture conditions and target genes crucial for genetic manipulation approaches. To achieve this aim, we developed the ‘Secondary Metabolite Transcriptomic Pipeline’ (SeMa-Trap). Available at https://sema-trap.ziemertlab.com, SeMa-Trap allows for efficient transcriptome mining of BGCs in bacteria through a user-friendly web interface. The pipeline performs RNA-Seq based transcriptome analysis of BGCs predicted by antiSMASH, compares their fold-changes in various experiments, and allows for promising experimental design and prioritization of the target genes for BGC overexpression. Finally, SeMa-Trap provides interactive result pages for each BGC. This allows easy exploration of BGC expression under certain culturing conditions and the identification of co-regulated genes, which may be located elsewhere in the genome and display potentially interesting functions as defined by the KEGG database (28). Here we provide an overview of the pipeline, highlight the visualization of the interface and demonstrate the efficacy of SeMa-Trap through a case study.

## MATERIALS AND METHODS

### Workflow

**Figure 1.**
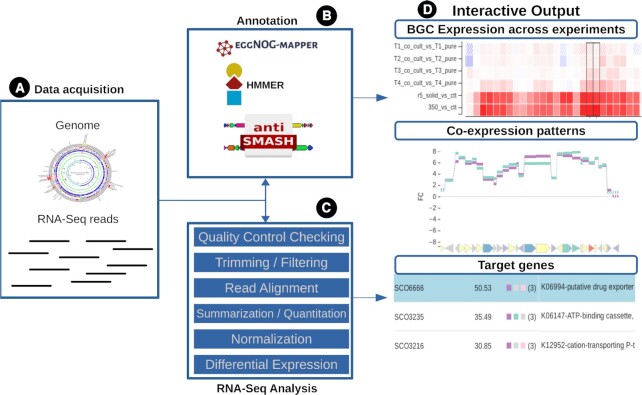
Overall workflow of the SeMa-Trap pipeline. First, the genomic and transcriptomic data provided by the user are acquired from relevant databases (**A**). Next step is the genome-wide annotation of the BGCs, essential housekeeping genes, secondary metabolite specific pathways and genes shown to have an impact on SM production (**B**). Final steps include a complete RNA-Seq analysis (**C**) and the generation of the interactive results (**D**).

The SeMa-Trap pipeline consists of 4 key steps (Figure 1). The first step is the acquisition of user provided genome and RNA-Seq data. Afterwards, genes involved in BGC expression regulation in the genome (e.g. transporters or regulators, referred to as genes of interest) are annotated, and BGCs are predicted by antiSMASH. BGC annotations in addition to those identified by antiSMASH can also be provided by the user by using the ‘Defined clusters’ option. To generate reference expression levels, essential housekeeping genes are also identified. In the third step, RNA-Seq analysis is performed to obtain expression levels and fold changes of the genes and BGCs of interest. Finally, results are presented by interactive visualizations and summarizing tables for easy exploration of the expression level changes. All results are kept in the server for 2 months. In addition, they can also be downloaded by saving the results page to the local machine. In case of larger data analysis, local installation and combining SeMa-Trap with in-house analysis pipelines is also possible using Anaconda.

### Input options and data acquisition

#### Input form

SeMa-Trap accepts user provided genomes in GenBank and FASTA format, however, the ideal input is the assembly accession number of the annotated GenBank file since that, in turn, will result in the automatic download of all annotation files from the NCBI FTP server. For efficient housekeeping gene identification, the corresponding taxonomic clade of the organism (e.g. Actinobacteria) should be selected through the ‘Reference set’ option. If the input genome is not represented by any available reference set, the ‘Unknown’ option offers HMM models acquired from the Database of Essential Genes (Supplementary Table S1) (29).

#### RNA-Seq data

For RNA-Seq based data options, allowed input types are run accession numbers from NCBI-SRA or EBI-ENA. Since it is imperative that the reads are downloaded in a fast and reliable fashion, SeMa-Trap utilizes multiple downloading options. IBM Aspera (https://www.ibm.com/products/aspera), a high-speed file transfer system, is the preferred and recommended way of data transfer (https://www.ncbi.nlm.nih.gov/books/NBK242621/). In case of any complications, SeMa-Trap will directly download from FTP servers or using fastq-dump (http://ncbi.github.io/sra-tools/). In case of pre-analyzed RNA-Seq data with other specific tools or parameters, the corresponding ‘BAM’ formatted files can also be uploaded. Limitations due to the current computational power and the implementation of the server are provided in the Supplementary Methods.

### RNA-Seq analysis

Once data acquisition is complete, SeMa-Trap utilizes several tools for analyzing the RNA-Seq data. Firstly, the fastp algorithm (30) is used to filter reads with low quality and for adapter trimming. Afterwards, filtered reads are mapped to the reference genome by Hisat2 (31) and sorted to generate corresponding BAM formatted files via samtools (32). Read count per gene is summarized by featureCounts (33). Finally, gene expression normalization takes place for each gene using the transcript per million (TPM) method described by Wagner et al. (34), and differential expression analysis is performed using DESeq2 (35), as detailed in Supplementary Methods. For the calculation of expression level or fold change of a BGC of interest, average expression of the ‘core biosynthetic genes’ (annotated by antiSMASH) is taken into account.

#### Scoring

In order to prioritize target genes, SeMa-Trap uses a scoring function dependent on the gene expression levels throughout the comparative transcriptomic experiments. To calculate such scores, fold changes of the selected BGC and the gene of interest are multiplied and then the calculated numbers from each selected experiment are added together (exemplified in Supplementary Table S2). However, it must be noted that a high score does not necessarily prove an association between a BGC and a gene. It rather points to high expression changes in the different conditions relative to a BGC of interest. Only when using large amounts of expression data, credible associations can be effectively detected (36).

#### Reference expression level

In order to set meaningful thresholds to label a BGC as ‘expressed’, SeMa-Trap uses three different average expression levels of specific genes. One of them is the mean expression of housekeeping genes throughout the genome. These genes are annotated by hmmsearch (37) with specific TIGRFAM models (38) unique for each reference set (39,40). The idea here is that on average, a gene defined as ‘essential housekeeping gene’ should be expressed significantly to be used as a reference for expression (41). However, BGCs can be expressed at lower levels and still produce compounds (42). Since no exact threshold exists to define BGC expression, SeMa-Trap offers separate reference levels such as the mean of non-housekeeping genes or all of the existing genes.

### Annotation

Apart from antiSMASH’s BGC prediction, the KnownClusterBlast algorithm is also applied to identify the compounds potentially produced by the BGC. If the provided genome is in FASTA format, an initial gene prediction step will take place using Prodigal (43). Since it is shown that certain types of genes actively control BGC expression, an extensive annotation of the genome is essential for prioritizing target genes to manipulate for BGC overexpression. For this purpose, the eggNOG-mapper (44) is used, particularly for the annotation of genes encoding transporters and genes residing in secondary metabolite specific KEGG pathways termed as ‘biosynthesis of secondary metabolites’ and ‘biosynthesis of antibiotics’. Using hmmsearch, genes conferring antibiotic resistance or genes with regulatory functions are further defined via specific HMM models procured from PFAM (45), Resfams (46) and CARD (47) databases.

## RESULTS

### Overview

**Figure 2.**
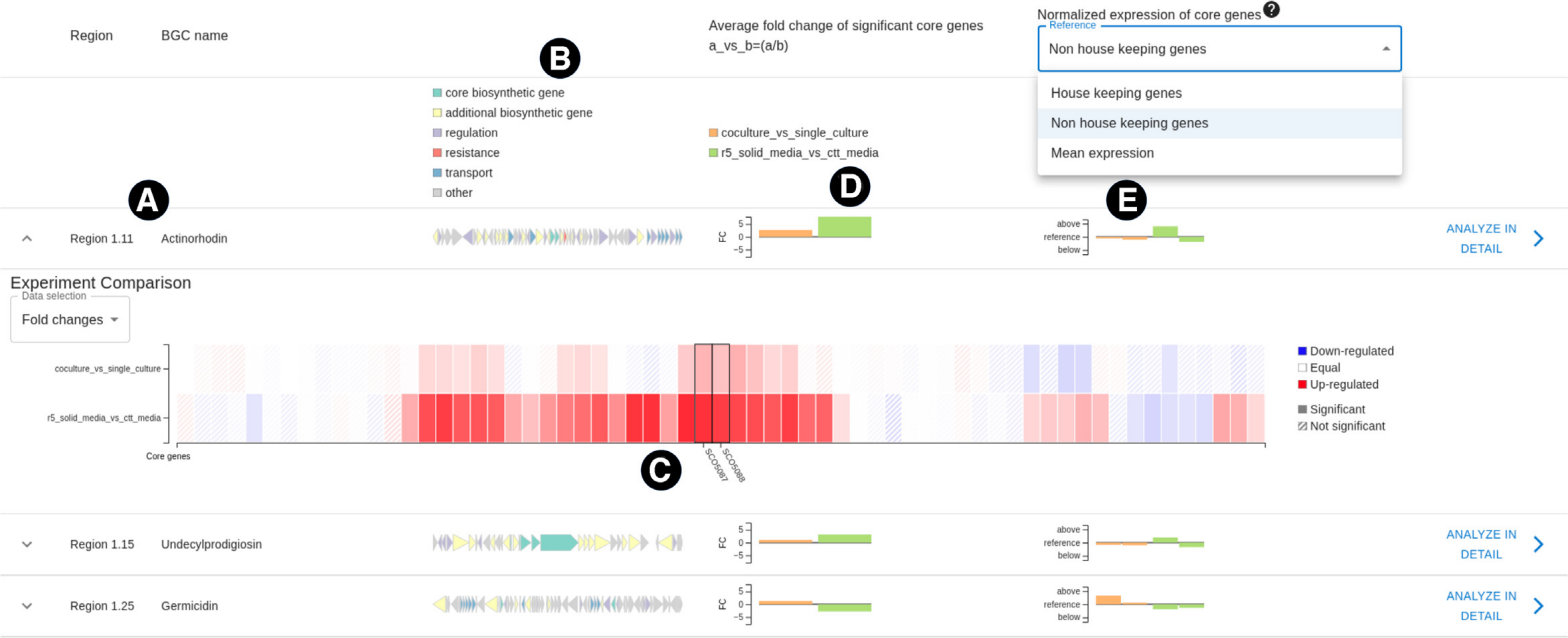
Overview result page of SeMa-Trap run for two comparative transcriptomic experiment designs. (**A**, **B**) The potential compound of the BGC and functional annotations of the genes within, respectively. (**C**) Heatmap of the BGC of interest, displaying each genes fold changes in different experiments. (**D**) Average fold change of the entire BGC, per experiment. (**E**) Expression (TPM) of a BGC relative to the selected, normalized reference expression level.

Once the analysis is complete, SeMa-Trap presents the overview of the overall fold changes of predicted BGCs and their expression levels relative to either of the mentioned reference expression levels (Figure 2). Various useful annotations of the genes in the BGCs are presented as well as the corresponding compound of the BGC if it is defined by KnownClusterBlast. Furthermore, a heatmap of the BGC content can be viewed in order to inspect fold changes of genes per experiment. BGCs can be further explored by clicking on the ‘Analyze in detail’ button.

### Case study

A recent study by Lee *et al.* demonstrated the various effects of microbial co-culturing on natural products biosynthesis at the transcriptome level (48). Using six different comparative experimental designs, the authors revealed that competition for iron increases the expression of specific genes leading to actinorhodin overproduction in *Streptomyces coelicolor* A3(2) when co-cultured with *Myxococcus xanthus*. In the following, by analyzing their publicly available RNA-Seq data, we illustrate how SeMa-Trap simplifies the entire analysis.

#### Visualization options and pathway analysis

**Figure 3.**
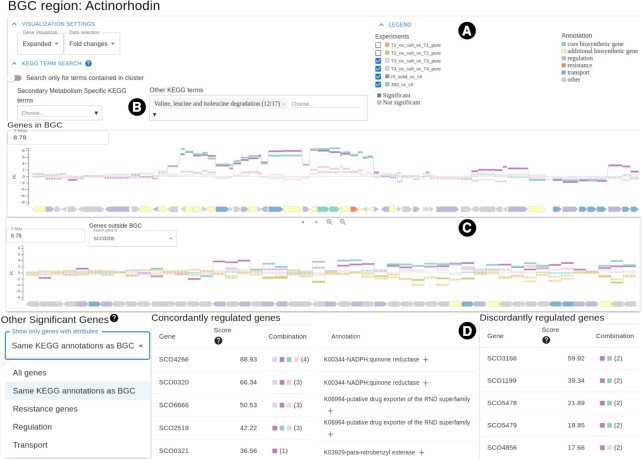
BGC centered results of SeMa-Trap. Initially, color codes for different annotations and multiple visualization settings are presented (**A**). Users can also highlight genes in specific pathways and choose to visualize the results based on the selected experiments (**B**). In section (**C**), two genome browsers are available in order to explore gene expressions from the selected experiments in the predicted cluster and throughout the genome. Finally, genes which are likely impacting the BGC expression based on transcriptomic data can be viewed through an interactive table **(D)**.

The first part of the result page (Figure 3A) offers a range of options such as various displaying options for the presented genes, the selection of specific experiments, and visualization of RNA-Seq results by fold change or TPM based expression level. Furthermore, it is possible to analyze specific pathways more in detail and explore the amount of differentially expressed genes within. In the presented case study, genes involved in the leucine and isoleucine degradation pathways were shown to be overexpressed, which potentially provide precursors for the actinorhodin biosynthesis. Using Sema-Trap this can easily be highlighted (Figure 3B).

#### Genome browser

For the investigation of specific genes within the BGC or throughout the rest of the genome, a dynamic genome browser is available. Apart from efficient exploration of gene expression and annotation, the genome browser offers multiple options. Provided that the BGC of interest is significantly expressed, it is possible to set more accurate boundaries for the predicted BGC. Within the antiSMASH defined boundaries of a BGC (Figure 3C), a smaller, continuous succession of genes appears to be co-expressed, suggesting that those are regulated in an operon and represent the actual BGC boundaries.

#### Target gene prioritization

After thorough investigation, Lee and colleagues identified the SCO6666 gene encoding a transport system alternative to the one in the actinorhodin BGC, which is encoded by the genes SCO5083–5084. Furthermore, they found that the SCO6666 gene highly affected the production of actinorhodin in iron restricted conditions. Such prioritization can be easily made using the SeMa-Trap tables sorted by concordantly and discordantly co-regulated genes including scores (Figure 3D). Selection of the functional category ‘Same KEGG annotations as BGC’ further simplifies the investigation of the systems alternative to those encoded within the BGC of interest. The ‘Combination’ column denotes the selected experiments, thus providing information on which genes are co-regulated with the BGC of interest under which conditions.

### Proof of principle

**Figure 4.**
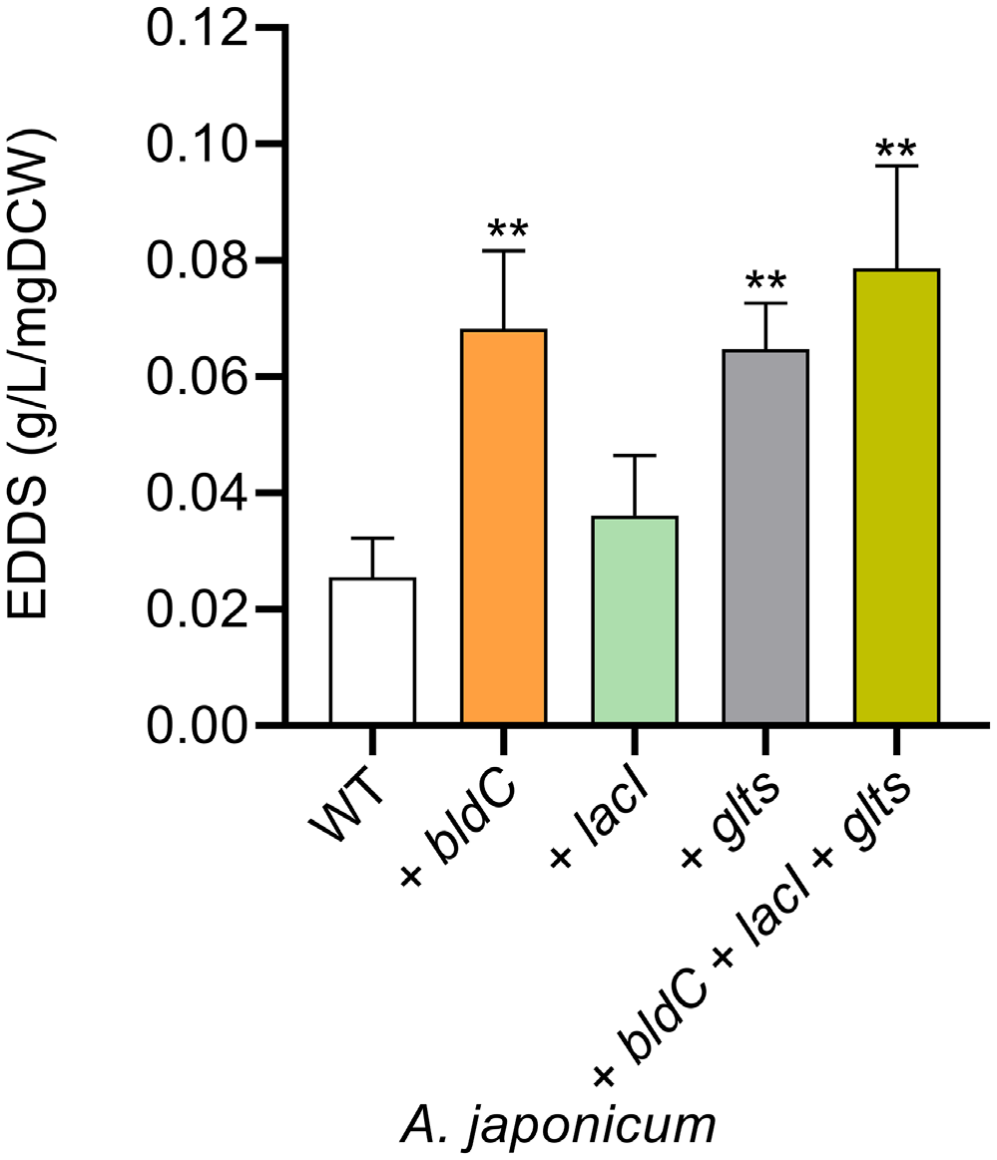
[*S,S*]-EDDS production in *A. japonicum* WT and recombinant strains. Strains were grown for 96 h in zinc depleted synthetic medium (SM). *A. japonicum* wild-type (WT); *A. japonicum* containing an additional copy of the genes *bldC*, *lacI* or glutamate synthase (*glts*), respectively and *A. japonicum* containing an additional copy of the three genes (*bldC* + *lacI* + *glts*).

As a proof of concept, we used SeMa-Trap to examine the transcriptome data of the actinomycete *Amycolatopsis japonicum*. *A. japonicum* is the producer of the complexing agent [*S,S*]-EDDS (49), a structural isomer of EDTA, which in contrast to EDTA is biodegradable and can replace EDTA in many industrial applications. However, [*S,S*]-EDDS production is inhibited by zinc at concentrations of 2 μM (50). Responsible for this regulation is the zinc uptake regulator *Zur*. To produce [*S,S*]-EDDS even in the presence of zinc the mutant *A. japonicum* Δ*zur* (referred to as zurko) was generated (51). To determine which genes to overexpress to increase [*S,S*]-EDDS production in *A. japonicum*, we performed transcriptomic analysis. For this purpose, RNA-Seq analyses of *A. japonicum* wild type (WT) and *A. japonicum* Δ*zur* cultured in the presence and absence of zinc for 24 h were performed. Thereby, a direct correlation between *zur* gene expression and the [*S,S*]-EDDS biosynthetic genes (BGs) could be observed. In particular, using SeMa-Trap we identified genes that exhibited high co-expression with the [*S,S*]-EDDS BG (concordantly regulated genes) and genes regulated in opposite manner (discordantly regulated genes). Since gene deletion is a multi-step, time-consuming process, we opted for a straightforward approach and overexpressed the targeted genes as a proof of concept. Thereby, we focused on genes with a regulatory function and those connected to secondary metabolism pathways. The target gene *bldC* (‘AJAP_RS36645’), with the second highest score in the category ‘regulation’, encodes a transcriptional regulator of differentiation which controls entry into development and the onset of antibiotic production in *Streptomyces* (52). The *lacI* gene, (‘AJAP_RS11995’), encodes a pleiotropic regulator (fifth highest score in the category ‘regulation’) which enhanced the production of antibiotics in *S. coelicolor* (53). From the pathways connected to secondary metabolism, we selected the glutamate synthase-encoding *glts* (‘AJAP_RS11230’) gene (with second best score) involved in glutamate biosynthesis. Since glutamate can be converted into L-aspartic acid, one of the precursors for EDDS biosynthesis, this gene was also taken into consideration. None of the selected genes have been experimentally shown to be linked to the [*S,S*]-EDDS production. Simultaneous overexpression of these genes resulted in an increased EDDS production by 3-fold compared to *A. japonicum* WT (Figure 4). Along with the experimental design, detailed methods (Supplementary Tables S3 and S4) and analysis (Supplementary Figures S1 and S2) can be further seen in the Supplementary Data.

## CONCLUSIONS AND FUTURE PERSPECTIVES

Leveraging on state-of-the-art sequencing techniques, comparative transcriptomic analyses have been continuously used to identify genes that are co-regulated with BGCs of interest and can be manipulated to activate silent BGCs. A variety of tools exists in order to annotate and effectively visualize biological functions of co-regulated genes such as KOBAS (54), conduct RNA-Seq analysis such as ProkSeq (55) or identify BGCs with co-expression data such as CASSIS (56). However, to the best of our knowledge, SeMa-Trap is the only public web server that combines genome mining and transcriptomic approaches for the identification of potential target genes for SM overproduction. The user-friendly graphical interface of the web server allows efficient and easy mining of RNA-Seq data, and was conceived for natural product researchers who are not acquainted with command line tools. Notably, SeMa-Trap also visualizes essential information about the cell response to the production of SMs on a transcriptomic level.

We showed herein that SeMa-Trap greatly facilitates the identification of co-regulated genes as illustrated on the actinorhodin-encoding BGC. However the limitations of the pipeline must be noted. The current scoring system is only designed to sort genes based on their similarity in transcription levels to a BGC of interest. It can not be used as an exclusive method for the selection of target genes. Thus, it is incumbent upon the users to further evaluate the hits returned by SeMa-Trap. For example, in the presented [*S,S*]-EDDS overproduction experiment, our literature search showed that the genes having the best co-expression score were unlikely to play a role in [*S,S*]-EDDS production. Consequently, three of the promising target genes were successfully overexpressed, leading to increased [*S,S*]-EDDS production. Especially when based on a few number of transcriptomic experiments, it becomes more likely that the SeMa-Trap analysis will include false positive target genes in the resulting tables. For future applications, by analyzing large amounts of publicly available RNA-Seq data, we are working on generating associations with certain gene types and classes of BGCs. Through co-expression networks, using statistical methods such as Pearson correlation coefficient, our aim is to reduce the number of false positives (57,58).

In summary, considering the ever-growing need for novel bioactive compounds, we believe that SeMa-Trap will serve as a helpful tool for the natural product community by facilitating the identification of specific co-expression patterns between different types of BGCs and genes with potential regulatory functions. Additionally, such analysis will also improve our ability to define expression thresholds above which the actual production of the encoded compound is observed. Last but not least, knowledge about the global cellular response to SM production may be the starting point to devise alternative strategies to optimize compound production and identify potential resistance mechanisms.

## DATA AVAILABILITY

SeMa-Trap is publicly available online at https://sema-trap.ziemertlab.com/ with no access restrictions. All of the source code is available on Bitbucket at https://bitbucket.org/mehmetdirenc/sematrap/. Source code for generating only the interactive HTML output is also available at https://github.com/Integrative-Transcriptomics/bgc-expression-viewer. Transcriptomic data files for EDDS overproduction and presented case study are available in the NCBI Bioproject database under the accession IDs PRJNA809550 and PRJEB25075, respectively.

## Supplementary Material

gkac371_Supplemental_File
