# Supplementary material for: Secondary Metabolite Transcriptomic Pipeline (SeMa-Trap), an expression-based exploration tool for increased secondary metabolite production in bacteria
Source: Nucleic Acids Res. 2022 May 17;50(W1):W682–9. doi: 10.1093/nar/gkac371 (PMC9252823; doi:10.1093/nar/gkac371)
Supplement: gkac371_Supplemental_File [file gkac371_supplemental_file.pdf]

SUPPLEMENTARY METHODS:

Table S1. Reference set specific HMM models used in housekeeping gene annotation

Reference Set	HMM Count
Chlamydiae	445
Group1	398
Group2	968
Tenericutes	321
Fusobacteria	650
Verrucomicrobia	776
Deinococcus-thermus	599
Alphaproteobacteria	1098
Betaproteobacteria	923
Spirochaetes	765
Firmicutes	1019
Bacteroidetes	860
Actinobacteria	664
Delta_Epsilon-proteobacteria	866
Group3	921
Cyanobacteria	882
Gammaproteobacteria	1478
Unknown	1568

Methods

Server Implementation

Hosted on highly scalable de.NBI cloud system, the SeMa-Trap web server runs on Ubuntu Linux (18.04.5 LTS) utilizing 2 TBs of hard drive space (1.5 in total available for a single run), 36 CPUs with approximately 1 to 1.4TB of RAM depending on the workload of cloud resources. Server-side application is based on Python3 Flask framework (https://flask.palletsprojects.com/) and Jinja2 templating language (https://jinja.palletsprojects.com) with JavaScript for user friendly input options. In combination, Redis (https://redis.io/), Nginx (https://www.nginx.com/), Gunicorn (https://gunicorn.org/) and Supervisor (http://supervisord.org/) tools are used for request handling and process control.

RNA-Seq Analysis

Initially, a gene expression value is taken into account if it has the assigned padj value < 0.05, and the gene is considered as a differentially expressed gene (DEG) if its absolute fold change is > 2. A

defined threshold (0.05) is set because of its wide acceptance as being "statistical significance" however, as such thresholds can be invalid for different experiments **(1)**, the cut-offs can be redefined by the user depending on the given data and how strict cut-offs are set in the experiment.

Target gene prioritization

In order to calculate the given scores, fold changes of the selected BGC and the gene of interest are multiplied and then the calculated numbers from each selected experiment are added together. A visualized example of the scoring of SCO6666 gene (with score 50.53, shown in Figure 3 D), encoding the alternate transport system for actinorhodin BGC, can be seen in **Table S2**. SeMa-Trap result can also be seen for the whole analysis at https://sema-trap.ziemertlab.com/results/51e8fb5d-99e3-489b-aa57-2aa5cd2cc1cd.

Table S2. Example for scoring method.

**Experiment**	**Actinorhodin Fold Change**	**SCO666 Fold Change**	**Multiplied**
T3_co_cult_vs_T3_pure	1.37320261041672	7.6	10.436339839
T4_co_cult_vs_T4_pure	2.682977920151415	6.41060810700008	17.199520006
r5_solid_vs_ctt	7.824020991896	2.92558252110573	22.889819059
Cumulative Final Score			~50.53

Strains, plasmids and oligonucleotides

The strains and plasmids are listed in **Table S3**. The oligonucleotides are listed in **Table S4.**

Media and culture conditions

Escherichia coli strains were grown in Luria broth medium **(2)** at 37°C and were supplemented with 100 µg ml^{-1} apramycin when necessary to maintain plasmids. Liquid cultures of *A. japonicum* were cultivated in 100 ml of R5 medium **(3)** in an orbital shaker (220 rpm) in 500-ml baffled Erlenmeyer flasks at 29°C. Liquid/solid media were supplemented with 100 µg ml^{-1} apramycin to select for strains carrying integrated antibiotic resistance genes.

Construction of the plasmids pRM4-*bldC*, pRM4-*lacI*, pRM4-*glts* and pRM4-*bldC-lacI-glts*

To construct the overexpression plasmids, *bldC* (AJAP_RS36645), *lacI* (AJAP_RS11995) and glutamate synthase (*glts*)(AJAP_RS11230) genes of *A. japonicum* were amplified via PCR with the primers listed in **Table S4** and purified using QIAquick gel extraction kit . The pRM4 vector **(4)**,

containing the constitutive promoter *ermEp**, was linearized with the restriction enzyme NdeI and purified. Using NEBuilder HiFi DNA Assembly cloning kit (NEB, catalog no.E2621S) the linearized pRM4 was ligated with each of the amplified genes. In addition the pRM4-*bldC-lacI-glts* was constructed containing all the three genes. The plasmids were confirmed by enzymatic digestion and sequencing, and integrated into the genome of in *A. japonicum* WT. These steps allowed the generation of the overexpression strains *A. japonicum*::pRM4-*bldC*, *A. japonicum*::pRM4-*lacI, A. japonicum*::pRM4-*glts* and *A. japonicum*::pRM4-*bldC-lacI-glts.*

[*S,S*]-EDDS production test

Liquid culture of *A. japonicum* WT and recombinant strains was performed in 100 ml volume to determine [*S,S*]-EDDS production according to **(5), (6)**. The optimized synthetic medium (SM) consisted of glycerol (25 g l^{-1}), $MgSO_4 \times 7\ H_2O$ (1.2 g l^{-1}), Ferric (III) citrate (60 mg l^{-1}), KH2PO4 (8 g l^{-1}), $Na_2HPO_4 \times 2\ H_2O$ (12 g l^{-1}) and sodium glutamate monohydrate (11.3 g l^{-1}), which was used as the nitrogen source. Pre-cultures were grown on a rotary shaker (120 rpm) at 29°C in complex culture medium (glycerol (20 g l^{-1}); soybean meal (20 g l^{-1}) at pH 7.5) in 50 ml volume for 48 h. A total of 5 ml of this pre-culture was used to inoculate 95 ml of SM. The cultures were grown for further 96 h before the [*S,S*]-EDDS production was analysed.

Detection of [*S,S*]-EDDS biosynthesis using HPLC-DAD

[*S,S*]-EDDS measurement was performed as described by **(6).** The analysis was carried out on a HP1090M liquid chromatograph equipped with a thermostated autosampler, a diodearray detector and an HP Kayak XM 600 ChemStation (Agilent). A total of 10 µl of samples were injected onto a Hypersil ODS column (125 × 4 mm, 3 µm) fitted with a guard column (10 × 4 mm, 3 µm; Stagroma) and analysed by isocratic elution with solvent A – acetonitrile (96:4, v/v) at a flow rate of 1 ml min^{-1}. Solvent A consisted of 20 mM Sorensen's phosphate buffer (pH 7.2) with 5 mM tetrabutylammoniumhydrogensulfate. UV detection was performed at 253 nm. For data analysis, Chemstation LC3D software Rev. A.08.03 was used. Commercial [*S,S*]-EDDS in solution (Sigma Aldrich) was used as standard.

Quantification

The HPLC analysis was performed from 1 ml supernatant. In order to determine the production of [*S,S*]-EDDS of cells, the [*S,S*]-EDDS concentration was divided by the dry cell weight (DCW). The [*S,S*]-EDDS production was expressed by (g/l/mg DCW).

Table S3: Bacterial strains and plasmids used in this study

Strain or plasmid	Description	Source of reference
A. japonicum MG17-CF17	[*S,S*]-EDDS producing wild-type	**(7)**
pRM4	pSET152 *ermEp** with artificial RBS, Apra^r	(4)
pRM4-*bldC*	pRM4 carrying *bldC* gene from *A. japonicum* WT	This study
pRM4-*lacI*	pRM4 carrying *lacI* gene from *A. japonicum* WT	This study
pRM4-*glts*	pRM4 carrying glutamate synthase (*glts*) gene from *A. japonicum* WT	This study
pRM4-*bldC-lacI-glts*	pRM4 carrying *bldC, lacI* and *glts* gene from *A. japonicum* WT	This study

Table S4. Oligonucleotides used in this study

Primer	Sequence (5´-3´)
Primers used for amplification of the *A. japonicum bldC* (AJAP_RS36645) coding region overlapping with pRM4 vector	
bldC_pRM4_F	CGACGGTATCGATAAGCTAGCCAGGGGAGGACCCAATGACCGCGACCATGGGCGGA
bldC_pRM4_R	GGGCTGCAGGAATTCGATATCAAGCTTAGATCTCATCAGACCTTGCGAGCGGGCTCG
Primers used for amplification of the *A. japonicum lacI* (AJAP_RS11995) coding region overlapping with pRM4 vector	
lacI_pRM4_F	GGGCTGCAGGAATTCGATATCAAGCTTAGATCTCATCATGCGGGGTACTCCTGGGTCGATTCG
lacI_pRM4-R	CGACGGTATCGATAAGCTAGCCAGGGGAGGACCCAATGTCGCTGGCGAAGGTGGCCC
Primers used for amplification of the *A. japonicum* glutamate synthase (*glts*) (AJAP_RS11230) coding region overlapping with pRM4 vector	
GS_pRM4_F	CGACGGTATCGATAAGCTAGCCAGGGGAGGACCCAGTGGCTGATCCGACGGGTTTCCTGA AGTACG
GS_pRM4_R	GGGCTGCAGGAATTCGATATCAAGCTTAGATCTCATCAGACCACCGCGAGCGGCA
Primers used for amplification of the *A. japonicum bldC*, *lacI*, *glts* coding region overlapping with pRM4 vector	

bldC_F_assem_pRM4	CGACGGTATCGATAAGCTAGCCAGGGGAGGACCCAATGACCGCGACCATGG GCGGAAGG
bldC_R_assem_pRM4	ACCTTCGCCAGCGACATTCAGACCTTGCGAGCGGGCTCGCT
lacI_F_assem_pRM4	CCCGCTCGCAAGGTCTGAATGTCGCTGGCGAAGGTGGCCCG
lacI_R_assem_pRM4	CCCGTCGGATCAGCCACTCATGCGGGGTACTCCTGGGTCGATTCGCG
GSsmall_F_assem_pRM4	CAGGAGTACCCCGCATGAGTGGCTGATCCGACGGGTTTCCTGAAGTACGAC
GSsmall_R_assem_pRM4	GGGCTGCAGGAATTCGATATCAAGCTTAGATCTCATCAGACCACCGCGAGCG GCAACG

Figure S1

Target genes with regulation attributes for [S,S]-EDDS overproduction. Note that, after a literature search, second and fifth most co-regulated genes were selected for overproduction experiments. Genes with putative regulatory domains were left out from our selection because their annotations were not strong enough to deduce an actual regulatory mechanism for a BGC. Entire analysis can be seen at https://sema-trap.ziemertlab.com/results/4985a8de-4c61-426f-8011-51b6aaa51350.

Other Significant Genes

Show only genes with attributes

Regulation

Concordantly regulated genes

Gene	Score	Combination	Relative position to cluster	Product
AJAP_RS15075	78.93	(2)	1503045	NAD(P)H-binding protein
AJAP_RS36645	37.78	(2)	6235770	BldC family transcriptional regulator
AJAP_RS13380	36.94	(2)	1111660	sugar isomerase
AJAP_RS21735	36.92	(2)	2994223	Gfo/Idh/MocA family oxidoreductase
AJAP_RS11995	30.92	(2)	757671	LacI family transcriptional regulator

Figure S2

Target genes that are defined in KEGG as specific to secondary metabolite "Metabolism" pathways for [S,S]-EDDS overproduction. Note that after a literature search involving precursor production mechanisms for [S,S]-EDDS, the second most co-regulated gene was selected for overproduction experiments.

BGC region: Edds

VISUALIZATION SETTINGS

Gene Visualizati... Expanded | Data selection Fold changes

KEGG TERM SEARCH

Search only for terms contained in cluster

Secondary Metabolism Specific KEGG terms: Metabolism (128/547) x Choose...

Other KEGG terms: Choose...

Concordantly regulated genes

Gene	Score	Combination	Relative position to cluster	Product
AJAP_RS15070	77.51	(2)	1501244	glycerol-3-phosphate dehydrogenase
AJAP_RS11230	48.42	(2)	604162	glutamate synthase subunit beta
AJAP_RS11225	46.35	(2)	599601	glutamate synthase large subunit
AJAP_RS21600	43.23	(2)	2966272	enoyl-CoA hydratase/isomerase family protein

References

(1) Jafari, Mohieddin; Ansari-Pour, Naser (2019): Why, When and How to Adjust Your P Values? In: *Cell journal* 20 (4), S. 604–607. DOI: 10.22074/cellj.2019.5992.

(2) Sambrook J, Fritsch EF, Maniatis T. 1989. Molecular cloning: a laboratory manual, 2nd ed. Cold Spring Harbor Laboratory, Cold Spring Harbor, NY

(3) Kieser T, Bibb MJ, Buttner MJ, Chater KF, Hopwood DA. 2000. Practical Streptomyces genetics. John Innes Foundation, Norwich, United Kingdom

(4) Menges R, Muth G, Wohlleben W, Stegmann E. 2007. The ABC transporter Tba of Amycolatopsis balhimycina is required for efficient export of the glycopeptide antibiotic balhimycin. Appl. Microbiol. Biotechnol. 77: 125–134.

(5) Zwicker, N., Theobald, U., Zähner, H., and Fiedler, H.P. (1997) Optimization of fermentation conditions for the production of ethylene-diamine-disuccinic acid by Amycolatopsis orientalis. J Ind Microbiol Biotechnol 19: 280–285

(6) Spohn, M., Wohlleben, W., Stegmann, E. (2016) Elucidation of the zinc dependent regulation in Amycolatopsis japonicum enabled the identification of the ethylenediamine disuccinate ([S,S] EDDS) genes. Environ. Microbiol., 18, 1249- 1263.

(7) Nishikiori, T., Okuyama, A., Naganawa, H., Takita, T., Hamada, M., Takeuchi, T., Aoyagi, T., Umezawa, H. (1984) Production by actinomycetes of (S,S)-N,N-ethylenediamine-disuccinic acid, an inhibitor of phospholipase c. J. Antibiot., 37, 426- 427
